# Conceptual and Operational Considerations in Identifying Socioenvironmental Factors Associated with Disability among Community-Dwelling Adults

**DOI:** 10.3390/ijerph120403814

**Published:** 2015-04-03

**Authors:** Mathieu Philibert, Robert Pampalon, Mark Daniel

**Affiliations:** 1 Département de Sexologie, Université du Québec à Montréal, Montréal, QC H3C 3P8, Canada; 2 Institut National de Santé Publique du Québec, Montréal, QC H2P 1E2, Canada; E-Mail: robert.pampalon@inspq.qc.ca; 3 Département de Médecine Sociale et Préventive, Université de Montréal, Montréal, QC H3N 1X9, Canada; E-Mail: mark.daniel@unisa.edu.au; 4 Département de Médecine Sociale et Préventive, Université Laval, Quebec, QC G1V 0A6, Canada; 5 Spatial Epidemiology and Evaluation Research Group, School of Population Health, Sansom Institute for Health Research, University of South Australia, Adelaide, SA 5000, Australia; 6 Department of Medicine, St. Vincent’s Hospital, The University of Melbourne, Fitzroy, VIC 3065, Australia

**Keywords:** disability, social participation, activities of daily living, residential characteristics, environments, socioeconomic inequalities

## Abstract

Disability is conceived as a person–context interaction. Physical and social environments are identified as intervention targets for improving social participation and independence. In comparison to the body of research on place and health, relatively few reports have been published on residential environments and disability in the health sciences literature. We reviewed studies evaluating the socioenvironmental correlates of disability. Searches were conducted in Medline, Embase and CINAHL databases for peer-reviewed articles published between 1997 and 2014. We found many environmental factors to be associated with disability, particularly area-level socioeconomic status and rurality. However, diversity in conceptual and methodological approaches to such research yields a limited basis for comparing studies. Conceptual inconsistencies in operational measures of disability and conceptual disagreement between studies potentially affect understanding of socioenvironmental influences. Similarly, greater precision in socioenvironmental measures and in study designs are likely to improve inference. Consistent and generalisable support for socioenvironmental influences on disability in the general adult population is scarce.

## 1. Introduction

Disability is conceptualised as situational difficulties arising from health conditions or impairments faced by individuals in performing daily living activities or social roles within a given social and built environmental context [1,2,3,4]. Conceptual frameworks thus situate disability at the interface of individual characteristics (e.g., functional limitations) and those of residential environments. Such an interactive definition of disability arose from critiques of individualised conceptions of disability. This allowed identifying enabling environments as targets for reducing disability [5,6,7]. Such strategies promote policies that address factors such as safety, accessibility of places, social support and equitable access to health and social services. Achieving the implementation of informed policies requires accounting for prior empirical evidence and the application of relevant theory.

An abundance of empirical studies have been published linking residential environments (*i.e.*, “neighbourhoods”) to various health-related outcomes [8,9,10,11,12,13]. In the 1990s and early 2000s, associations with neighbourhood characteristics began to be widely disseminated with the growing use of multilevel analysis, which enables differentiating compositional effects (*i.e.*, geographical clustering of individuals’ characteristics) from contextual effects (*i.e.*, milieus’ attributes) for explaining the spatial patterning of health [14]. Characteristics of residential environments are now considered to be important determinants of health inequalities [15,16,17,18]. However, relative to the magnitude and breadth of research on the influence of neighbourhood factors and health-related outcomes, few epidemiological studies have been published on the relationship between neighbourhood factors and disability.

More than a decade and a half ago, Stuck *et al.* [19] reviewed longitudinal studies reporting on risk factors for functional decline and disability. They found no studies assessing the effects of the built environment specifically and identified 21 studies addressing social factors. More recently, Clarke and Nieuwenhuijsen [20] and Yen *et al.* [21] reviewed the literature on environments and healthy ageing generally, encompassing health outcomes such as self-rated health and mortality. To our knowledge, no review targeting epidemiological analyses of neighbourhood factors associated with disability in the general population has been published since the review by Stuck *et al.* [19]. We conducted such a review, and report it here.

Our aim was to portray the epidemiological literature analysing socioenvironmental influences on disability. Our review focuses broadly on disability in the general community-living adult population and does not restrict itself to longitudinal studies. We do, however, restrict our focus to quantitative analyses. We use the term *socioenvironmental* for describing the built, social and attitudinal characteristics of residential neighbourhoods, variously defined. For this review, given the lack of consensus in operationalising disability [22], we defined disability as any kind of health-related constraint or impairment encountered in performing any type of daily living activities or social roles, including all areas of functioning (e.g., leisure, work or social functioning). We employ the term *disablement process* in reference not to any particular model but rather, for describing generally the genesis of disability.

## 2. Methods

Searches were conducted in Medline, Embase and CINAHL databases and were restricted to English or French language articles published in peer-reviewed journals between 1 January 1998 and 1 December 2014. For each database, a query was generated by mapping the following keywords to the corresponding entry in each database’s thesaurus: (disability OR activities of daily living OR handicap OR functional limitation OR participation) AND (spatial OR geography OR environment OR milieu OR neighbourhood OR urban OR suburban OR rural OR area OR local) AND (causality OR risk factors OR epidemiological factors OR socioeconomic factors). Synonyms were not used as they are considered by the thesauruses (*e.g.*, *disabled* and *disability* correspond to the same entry in PubMed’s thesaurus). Papers were then assessed for inclusion by one of the authors (MP) by reviewing titles and abstracts using the following criteria: (i) the target population was the general population of community-living adults; (ii) disability was treated as an outcome, using individual- or aggregated-level data; and (iii) a spatially-based socioenvironmental factor was modeled as a predictor of disability.

The first criterion was relaxed to allow for studies of gender- and age-defined population in order to avoid obtaining a biased representation of the literature. Studies were excluded if they used geography only for stratification or did not publish an estimate for the association between disability and a socioenvironmental factor (*i.e.*, controlling for such a factor without reporting the estimate). Reviews and meta-analyses were also excluded. References of included papers were assessed for inclusion. The selection of articles did not meet all the requirements for systematic reviews. This study is a narrative literature review.

## 3. Results

Our searches yielded 12,346 articles, of which 35 met the three inclusion criteria [23,24,25,26,27,28,29,30,31,32,33,34,35,36,37,38,39,40,41,42,43,44,45,46,47,48,49,50,51,52,53,54,55,56,57]. This article base was then supplemented with an additional eight articles [58,59,60,61,62,63,64,65] identified in the references of the 35 articles identified in our searches. Diversity in conceptual and methodological approaches was evident to the extent that studies could not be straightforwardly compared and contrasted. Characteristics of the 43 reviewed studies are presented in Table 1.

**Table 1:** Study characteristics.

Study Design	Age group of Target Population	Reference #	Authors, Date	Country	Sample Size
Cross-sectional	Entire population (census-based)	[50]	Philibert *et al.*, 2013	Canada	7,075,835 (census-based)
	Adults (≥ 16 years)	[60]	Feldman & Steptoe, 2004	UK	636
		[64]	Robert, 1998	US	3617
		[63]	Reijneveld, 1998	Netherlands	5121
		[49]	Philibert *et al.*, 2013	Canada	34,416
		[37]	Fuller-Thomson & Gadalla, 2008	US	645,835
		[38]	Gadalla & Fuller-Thomson, 2008	US	1,973,766 (census-based)
	Adults without elders (≥18 and <65 years)	[42]	Jongeneel-Grimen *et al.*, 2011	Netherlands	40,213
		[24]	Auchincloss & Hadden, 2002	US	176,930
	Middle-aged and older adults (≥40 years)	[33]	den Ouden *et al.*, 2013	Netherlands	537
		[30]	Clarke *et al.*, 2008	US	1195
		[32]	Clarke *et al.*, 2011	US	1225
		[56]	Yeatts *et al.*, 2013	China	1267
		[35]	Fogelholm *et al.,* 2006	Finland	2850
		[36]	Freedman *et al.*, 2008	US	15,480
	Older adults (≥65 years)	[47]	Morala, Shiomi & Maruyama, 2006	Philippines	200
		[53]	Richard *et al.*, 2012	Canada	520
		[45]	Levasseur *et al.*, 2011	Canada	554
		[43]	Kabir *et al.*, 2001	Bangladesh	696
		[28]	Bowling & Stafford, 2007	UK	786
		[58]	Bowling *et al.*, 2006	UK	999
		[39]	Giraldez-Garcia *et al.*, 2013	Spain	1106
		[51]	Rahkonen & Takala, 1998	Finland	1448
		[61]	Goins & Mitchell, 1999	US	1911
		[55]	Wight *et al.*, 2008	US	3442
		[59]	Clarke & George, 2005	US	4154
		[65]	Zimmer & Kwong, 2004	China	20083
		[26]	Beard *et al.,* 2009	US	937,875 (census-based)
		[62]	Lin, 2000	US	8% of US population ≥65 years (≈2,500,000; census-based)
Longitudinal	Entire population (registry-based)	[23]	Aida *et al.*, 2013	Japan	29,374
	Middle-aged and older adults (≥40 years)	[34]	den Ouden *et al.*, 2013	Netherlands	478
		[25]	Balfour & Kaplan, 2002	US	883
		[27]	Beydoun & Popkin, 2005	China	976
		[31]	Clarke, Ailshire & Lantz, 2009	US	1821
		[57]	Zimmer, Wen & Kaneda, 2010	China	2944
	Older adults (≥65 years)	[54]	Starr, Deary & Macintyre, 2003	UK	201
		[52]	Rantakokko *et al.,* 2009	Finland	993
		[29]	Clark *et al.*, 2009	US	1884
		[46]	Liang, Liu & Gu, 2001	China	2115
		[48]	Pérès *et al.*, 2005	France	3198
		[44]	Lang *et al.*, 2008	UK	4148
		[41]	Gu & Xu, 2007	China	6132
		[40]	Glymour *et al.*, 2010	US	10,273

### 3.1. Conceptual Bases

Thirteen studies referenced an explicit conceptual framework specific to disability [25,27,30,31,33,34,43,45,48,49,50,52,59]. Balfour & Kaplan [25] positioned their disability measure in relation to Nagi’s model [2] without hypothesising a specific pathway. Five studies [30,31,33,34,50] referred to the International Classification of Functioning, Disability and Health (ICF) [3]. In defining disability as a person–context interaction, Levasseur *et al.* [45] referred to the Disability Creation Process [1], Philibert *et al.* [50] referred to the latter model but also to the ICF and Verbrugge and Jette’s model [4], whereas Rantakokko *et al.* [52] referred to Verbrugge and Jette and to Lawton and Nahemow [66].

Seven studies [27,43,48,49,50,52,59] referred to Verbrugge and Jette’s model. Among these, four studies [48,49,50,59] postulated socioenvironmental factors as influencing the pathway from functional limitations (*i.e.*, individual-level) to disability. Two reports [27,43] referred to Verbrugge and Jette’s model for defining their disability measure but only one [43] was specific regarding how sociocultural and structural contexts might determine disability (*i.e.*, by influencing norms). One other study [49] referred to Verbrugge and Jette’s model for defining disability but referred to Glass and Balfour [67] for describing categories of socioenvironmental factors potentially influencing disability.

Other studies were not based on any disability-specific conceptual model. Many considered disability as an indicator of health or morbidity [24,37,38,39,42,44,56,57,63]. Most justified their analyses by presenting possible explanations for the differential distribution of disability according to socioenvironmental factors, drawing on previously published empirical reports.

### 3.2. Disability Measures

We found nearly as many measures of disability as there were reviewed papers. Two studies drawing on questions from the US census used different questions [26,62]. Three other studies [24,49,50] used general survey questions regarding limitations in daily activities with varying levels of precision in referring to activities. Four studies integrated aspects of social roles and interactions [28,32,45,53]. A majority of studies used disability measures that pertain to the domains of activities of daily living (ADLs) and/or instrumental ADLs (IADLs), though with variable operational forms. These studies predominantly involved elderly people and were almost equally divided between those measuring the activities in terms of individual’s ability (“can do”) and those measuring the actual performance (“do do”). The most common activities surveyed were those related to self-care and mobility. Mobility-related activities are the most frequently measured and exemplify well the large diversity of ways by which similar activities are measured.

Apparently similar activities were evaluated using different operational forms of activity. For example, walking was evaluated in terms of survey questions referring to (i) various objective distances (50 feet [47], quarter mile [25], half mile [29,48], 100 yards [46], 200 m [57], 400 m [52], and 100 m, 500 m, and two km [35]); (ii) various subjective distances (across a room [27,40,55], inside the home [37,38,39], several blocks [30,31,36,64], and outside the home [39,62,65]) or intensities (“running or jogging about 1 km” [56]); (iii) length of time spent walking (5 min [51]), and (iv) without any distance anchor whatsoever (simply *walking* [33,34,42,61]). Further, qualitatively different levels of precision were used in referring to apparently identical activities, such as *dressing* [28,36,61], “flexibility for dressing” [58] (p. 477) and “donning and removing a jacket” [47] (p. 102).

The ways that activities were integrated into a single measure varied between studies. Some authors referred to overlapping domains with different questions. For example, two studies assessed fine motor skills using two questions (one question for cutting toenails and another for tying shoelaces) [28,58] while the overall dimension represented by these abilities was covered by a single statement in another study (one question for writing or handling small objects) [26]. Furthermore, measures did not cover the same range of activity domains and the number of activities assessed varied between studies. For example, some asked about ADLs including “bathing, clothing, eating, *grooming*, transferring, toileting and walking across a small room” [27] (p. 2047) while others asked about “getting up from bed, going to toilet, bathing, eating, dressing and undressing, mobility indoors, *mobility outdoors*, and *taking medicine*” [43] (p. 360).

Most studies expressed disability dichotomously (with various cut points applied over a variable number of activities). Some used multi-category measures [30,58,64], others used interval scales of varying ranges [47,54,60], and some used a count of the number of dimensions in which a difficulty/limitation was reported [28,34,56,59,61]. In two studies [45,53], a disability measure was derived from the translation of categorical frequencies of performed activities (almost every day, at least once a week, at least once a month, less than once a month, never) into the sum of numbers of days for which disability was experienced.

### 3.3. Study Designs and Analyses

Twenty-nine papers involved cross-sectional analyses and 14 involved longitudinal analyses (Table 1). Unfortunately, variability in operational forms of disability measures and socioenvironmental factors (see below) precludes our comparing findings in terms of study designs. Further, given the dynamic nature of the disablement process over time [68,69,70], length of observation period was another factor affecting comparability. Among the 14 longitudinal analyses reviewed, one had a 15-year follow-up [31], one had a 14-year follow-up [40] three had 10-year follow-ups [34,48,54], one had an eight-year follow-up [29], and eight had follow-ups ranging from one to four years [23,25,27,41,44,46,52,57].

Different statistical procedures were used to estimate associations with socioenvironmental factors. A majority of studies used logistic or multinomial regression for modelling dichotomous or categorical outcomes. Linear regression was used by six studies modelling continuous outcomes [26,28,45,47,61,64]. Count data were modeled with Poisson [34,59], negative binomial [56] or linear [28,61] regressions. Multilevel models were used by 15 studies [23,28,30,32,36,37,38,41,42,49,50,55,57,59,63], mostly for estimating contextual effects above those of individual characteristics, but also by one study for estimating individual changes over time [31]. Hence, a majority of studies used individual-level models, thereby attributing socioenvironmental characteristics to individuals. One study did not specify the type of model [54] and the remainder used other types of models.

Fifteen studies analysed modification by socioenvironmental factors of an association between a personal attribute and disability. Stratification was used to assess modification by age and individual-level SES [29], ageing (time) [52], gender [23,41,45,49] or functional status [50]. Regression models were used for testing interactions between a given socioenvironmental factor and age [29], ageing (time) [31], individual-level SES [29,65], impairments or functional limitations [30,32,45,50,59], or between socioenvironmental factors [26,28,49]. Only two studies tested mediation [46,60].

### 3.4. Socioenvironmental Factors

Rural-urban difference and area-level socioeconomic status (SES) were the most frequently analysed socioenvironmental factors. Other socioenvironmental influences included residential stability [26,36,49,50], population density and demographic composition [26,31,33,34,36,44], crime or safety [25,26,28,29,32,36,39,52,58], income inequalities [37,38], traffic and street conditions [25,26,28,32,36,39,58], housing quality [49,50], land use [26,59], walkability and commuting [49,53], access to services [25,28,32,39,45,50,53,54,56,57,58], a composite index of physical and social disorder [32], aspects of social cohesion and/or social capital [23,26,28,39,53,54,60], and pollution and peacefulness [39].

Eleven of 18 studies examining rurality expressed this measure dichotomously [27,41,43,44,46,47,48,52,57,62,65] and other studies represented an rural-urban gradient via use of more than two categories [24,28,33,34,35,49,61]. Only nine studies used explicit criteria by which to define the urban–rural categorisation, these including: level of urban influence [24,49], population size [48,61], population density [28,33,34], and a combination of population density and distance to urban centre [35]. Others did not describe the criteria that underlay their classification system.

Eighteen studies analysed socioenvironmental influences using socioeconomic factors. Composite indices were used by 10 studies [26,28,31,36,40,42,44,49,50,55]. Other papers analysed single socioeconomic variables describing neighbourhoods in terms of characteristics such as income [24,37,38,56,57], occupation class [60], employment level [63,64], and poverty threshold [63,64]. Among the studies reporting on associations between disability and a measure of area-level SES, only two studies [31,57] reported the absence of such an association. However, six studies [24,28,40,55,63,64] reported that a statistically significant association between area-level SES and disability became non-significant after individual-level factors were accounted for.

While most studies used socioenvironmental measures derived from individual-level data, some used individuals’ perception [23,25,28,29,39,45,52,53,54,57,58,60] and others used measures of area-level, non-individual features [26,30,32,36,50,53].

Notwithstanding studies which measured socioenvironmental factors on the basis of perceptions at the individual level, socioenvironmental factors were used to characterise geographical areas of varying sizes. Twenty-seven studies expressed socioenvironmental factors based on political or municipal definitions (e.g., counties, cities, or countries) or administrative spatial units (e.g., census tracts, or enumeration districts). Others did not provide a description of the spatial units used. Ten studies explicitly addressed the choice of geographical scale for representing socioenvironmental factors [24,29,30,31,36,44,49,50,59,64] and most of these studies acknowledged the arbitrariness of the spatial delineation used.

Many studies reported significant associations between socioenvironmental factors and disability. Among these, a large proportion observed such an association while accounting for individual-level factors. However, almost half of the reviewed studies also reported negative results. Unfortunately, variability in underlying concepts and in operational and analytical choices makes difficult an assessment of trends in associations between disability and socioenvironmental factors (except for area-level SES).

## 4. Discussion

This review indicates that a variety of socioenvironmental factors have been identified as associated with disability. Among such influences are area-level SES and rurality. In spite of the diversity in concepts, measurements, populations and study designs, the studies reviewed here indicate that area-level SES is inversely associated with disability. Studies using rurality measures observed inconsistent results.

This review clearly demonstrates the diversity of underlying concepts and operational choices. The diversity of conceptual bases (*i.e.*, constructs and their relationships), disability measures, socioenvironmental factors evaluated and methods used, including different designs, yields little basis for systematically comparing studies. The results of this review highlight several issues that influence estimations of socioenvironmental effects: concepts and measures of disability, constructs and measures of the environment, and study designs and statistical analyses.

### 4.1. Conceptualising and Measuring Disability

Few studies referred explicitly to a conceptual model of disability and, of those that did, disability was not equally operationalised, nor were associations with socioenvironmental factors consistently tested. Some studies considered their outcome as a measure of health status. The distribution of a disability measure in a population will vary according to the underlying conceptual definition and operational form used [71], thus affecting any measured association with other factors. Consequently, the variability of disability measures encountered in this review impacts the extent to which associations between socioenvironmental factors and disability can be compared.

This review showed that the word *disability* conveys different meanings, either intentionally or unintentionally. This could partly be explained by the persistence of a biomedical representation of disability inherited from WHO’s former classification [72] which tends to maintain an individualised conception of disability as a marker of health, as opposed to interactive definitions of disability proposed in more recent models. Another explanation could reflect difficulties encountered in defining some activities as being either context-sensitive or context-free (person-level) [22]. The latter challenge especially impacts measures of ADLs.

A conceptual positioning of operational measures of ADLs can be undertaken by applying Rose’s thesis [73]. Rose juxtaposed the causes of individual cases with the determinants of the population incidence rate. In so doing, he illustrated that any capacity to detect influential factors depends on the level of observation (*i.e.*, the causes of disease ascertained by comparing individuals within a population may not correspond to the basis of differences in incidence rates ascertained through contrasts of populations). A corollary is that at a given level in a multilevel causal system, only certain determinants will have a uniform effect while others will be heterogeneous. In population health studies, defining the target population through defining the study area is akin to choosing a level of observation, that is choosing a “tacit causal field” [74]. The larger the study area, the greater the heterogeneity of social and cultural factors affecting the distribution of daily activity patterns in the population. Hence the term “neighbourhood” whilst providing for ease of framing “residential-area” characteristics corresponds in the literature to a large breadth of spatial definitions of populations and/or areas.

Badley [75] proposed that homogeneously distributed socioenvironmental factors may act as “scene-setters” that influence one’s activities irrespective of one’s health. Thus, ADLs could be potentially be viewed as context-independent (serving as markers of person-level abilities) for collectives of individuals homogeneous in their exposure to a given socioenvironmental factor influencing daily activities (e.g., a cultural trait). Otherwise, and most likely, ADLs could be context-sensitive although an underlying need for executing ADLs could be highly homogeneous, depending on the extent of the study area. Many of the reviewed studies used measures of ADLs, some describing individuals’ abilities and others describing individuals’ performance of activities. Positioning of measures of ADLs as operational forms of person-level abilities (context-free) or disability (context-dependent) will lead to fundamentally different inferences in terms of socioenvironmental influences in the disablement process.

This review identified two other important operational issues relevant to quantitative analyses of associations between disability and socioenvironmental factors: the set of activities considered in a disability measure and how many activities are integrated into a single measure.

One aspect of the operational form of a disability measure is the set of activities that it encompasses. For example, four of seven reports referring to Verbrugge and Jette’s [4] model [27,43,48,59] operationalised their outcome using ADLs and IADLs; however, each did so differently, assessing different sets of activities. Two included the use of public transports in IADLs while the two others did not. Use of public transport can be associated with places’ characteristics (e.g., transit system network and distances) differently from other activities. As not all activities are equally associated with the same socioenvironmental influences, these choices affect the sensitivity of a disability measure to socioenvironmental factors (all else being equal).

Consistent with the findings of Stuck *et al.* [19], this review found conceptual inconsistencies in some disability measures. In some cases, various activities conceptualised as different constructs of the disablement process were integrated into a single disability measure. For example, one study used an indicator that combined, among others, measures of bending, reaching, getting on a bus and shopping [54]. Such conceptual overlapping (*i.e.*, the combination of person-level capacities and of performance in context-sensitive activities) reduces any potential to situate the socioenvironmental influences in the disablement process. The possibility of pinpointing socioenvironmental effects is further affected by the fact that these multi-dimensional measures are often dichotomised.

The type and number of activities integrated into a disability measure as well as how they are integrated will influence the observed association between disability and socioenvironmental factors. This illustrates the importance of having an *a priori* conceptualisation of the relationship between socioenvironmental factors and disability. For meaningful inferences to be made on the latter, it is essential to isolate socioenvironmental influences from personal attributes. Analyses therefore require operationalisations that provide for distinguishing between individuals’ capacities (context-free) and performance in context-sensitive activities (*i.e.*, “can do *vs.* do do”) [1,4]. Further, the assessment of performance in context-sensitive activities requires accounting for one’s need (or willingness) to perform a given activity, irrespective of the presence of socioenvironmental obstacles or facilitators. Non-performance does not necessarily result from the influence of a socioenvironmental obstacle (*e.g.*, public transportation may not be used even if fully accessible).

### 4.2. Conceptualising and Measuring Socioenvironmental Factors

Contrarily to the review by Stuck *et al.* [19] our review identified analyses which dealt with features of the built environment. This finding may reflect the increased interest in the general epidemiological literature for such factors as well as developments in conceptualising disability as a person-context interaction. However, many of the reviewed papers used socioenvironmental measures encompassing various socioenvironmental factors, thereby leading to results potentially difficult to translate into interventions on specific socioenvironmental features. Precision in the choice of the indicators used can inform an understanding of the process by which they operate.

The ontological definition of socioenvironmental factors is of primary importance for conceiving how the residential area or neighbourhood milieu interacts with individual attributes in leading to individuals experiencing disability. A milieu can be conceptualised as a set of spatially-based attributes causally associated in the production of an observed response [76]. Many characteristics of places can influence disability, in various ways. Therefore, coarse operationalisations of socioenvironmental characteristics (e.g., “rural”, which encompasses various compositional and contextual factors) can lead to estimating overall effects that are unrepresentative of any of a multitude of specific influences. A large proportion of the papers reviewed here evaluated the effect of rurality using coarse indicators, thereby implicitly assuming a global, non-specific influence of this milieu.

Drawing from research on accessibility (e.g., Imrie [77]) and from analyses of a variety of health outcomes (e.g., Paquet *et al.* [78] and Weich *et al.* [79]), epidemiological research on disability should seek to integrate precise measures of features of the physical/built environment which may affect daily activities, *e.g.*, housing quality, and accessibility to local and health services, public spaces, and transportation. A lack of detail on such specific influences does not assist policymakers who need to know the target and potential gain for intervention on socioenvironmental factors. Depending on factors integrated, composite indicators may also be considered to be coarse operationalisations. Assembling *correlated factors* into a single measure does not ensure a description of *equal processes* by which socioenvironmental factors influence disability. Caution is necessary when choosing factors to be integrated within a single measure to ensure precise estimation and relevant inference. The identification of global trends is a useful and necessary step in many scientific investigations, opening the door to more detailed analyses of precise socioenvironmental factors. The risk, however, is in potentially concluding that there is an absence of socioenvironmental effects after having observed a null effect for a global indicator that could mask opposing trends. At the least, combinations of factors representing different processes can under- or over-estimate specific socioenvironmental influences.

Many of the reviewed studies measured socioenvironmental factors using aggregated data. Whether for assigning area-level characteristics to individuals or for modelling their influence at a higher level, most papers reviewed here had utilised areas defined by administrative authorities (including official statistical offices) without justifying their selection (administrative data are often available at various scales). Only 10 studies discussed the choice of geographical scale and in so doing predominantly conceded that the arbitrarily-defined area utilised might not have been the most appropriate operationalisation for the analysed socioenvironmental influences. Geographical scale is likely to affect associations with disability by influencing the level of heterogeneity of the distribution of socioenvironmental variables. This relates to the modifiable areal unit problem (MAUP): different regression coefficients can be obtained using the same data set by varying the number of spatial units (*scale effect*) and the delineation of their boundaries (*zonation effect*) [80,81].

Krieger *et al.* [82] showed that using the same cancer incidence data, rate ratios can be 0.94, 0.91 or 1.33 depending if the socioenvironmental factor (percentage of homes in high-end price range) was calculated over block groups, census tracts or postal code areas, respectively. The difference between considering a socioenvironmental factor as being protective or not is of enormous relevance to policy making. Unfortunately, we do not know how much this example is extreme since the choice of the spatial unit is rarely explicitly rationalised in terms of the theoretical premise of a study, including those papers reviewed here. In multifactor, hierarchical causal systems, it is unlikely that all determinants will operate, interactively or not, to produce the same spatial patterning of disability. The idea that “one boundary set fits all” is therefore unrealistic, but implicitly accepted through the frequent use of a single administratively-defined boundary set for modelling different socioenvironmental factors.

Socioenvironmental factors were measured through individuals’ perception in many of the reviewed studies. Using subjective or objective measures for operationalising socioenvironmental factors will affect consequent understanding of their contribution to the disablement process. Each type of measure corresponds to specific pathways. In effect, given that an individual’s perception is likely to mediate the influence of socioenvironmental obstacles or facilitators, both objective and subjective evaluations of socioenvironmental features are required to more fully understand their influence. Insight on pathways also depends on study design and analyses.

### 4.3. Study Designs and Analyses

The disablement process is made of complex trajectories in which transitions (*i.e.*, decline and improvement) occur, with varying transitional patterns occurring depending on risk factors and individuals’ capacities [68,69,70]. Longitudinal or cross-sectional data categorise a population differently (incident cases are not the same as prevalent cases), thus capturing different temporal and underlying dynamics. While incidence data reflect disability onset and potentially transitions, prevalence data will be sensitive to incidence rate and disability duration and also disability consequences (e.g., change in place of residence following modification of individuals’ SES or migration for accessing required health services). Thus, how a study design integrates temporality (*i.e.*, duration of the follow-up period and frequency of observations) will determine the dynamics to which a disability measure is sensitive and therefore, impacts on its association with socioenvironmental factors.

The analytic framework is another aspect of study design that will affect the portrait of socioenvironmental influences on disability. The notion of person-environment interaction seemed to be acknowledged (at least implicitly) by many of the studies reviewed here. Some also evaluated relationships between socioenvironmental factors and disability, in terms of mediation mechanisms. However, in the majority of reviewed papers, analyses tested for the “independent” contributions of socioenvironmental factors, simply accounting for individual-level covariates in testing the main effects of socioenvironmental predictor variables.

Clarke and George’s [59] results illustrate well the importance of using an appropriate analytic framework: housing density and land-use diversity were not significant independent predictors of disability but their interaction with functional limitations was statistically significant. This demonstration was possible because the analytic framework underpinned a hypothesis of effect modification by socioenvironmental factors on the pathway from functional limitations to disability. For that matter, calls for considering variability in individuals’ susceptibility to socioenvironmental characteristics have also been made for research on health outcomes [83,84]. Such specificity is of paramount importance for unravelling “the black box of places” [85], especially for understanding their contribution to the interactive phenomenon which is disability.

Socioenvironmental characteristics are also conceptualised as determinants of disability risk factors and thus, their influence on disability may be mediated by individual-level factors. Detailed conceptualisation of specific pathways and exploration prior to analysis of the qualitative assumptions underlying these pathways can help in understanding the interplay between individual and socioenvironmental characteristics, namely, distinguishing between confounders and intermediate variables [85]. The choice of control variables is also susceptible to inform on how place effects operate. Many reviewed studies reported socioenvironmental associations with disability as being sensitive in controlling for individual-level markers of SES. Pérès *et al.*’s [48] found living in a rural area to be a significant predictor of recovery from disability but that effect disappeared after controlling for pathologies, impairments and other risk factors, suggesting an influence on disability operating through pathologies and impairments or confounding by risk factors. In this case, however, the analytic framework did not allow for distinguishing between mediation or confounding.

The association between individuals’ and places’ characteristics raises issues of confounding and, potentially of, over-adjustment via statistical control. The pitfall of over-adjustment is illustrated by one reviewed study [51]: effect of rurality was estimated while simultaneously controlling for individual SES using an indicator of occupation in which “farmers” was contained as a class.

Confounding between individual and socioenvironmental factors used in explaining the spatial patterning of health has led to an increased use of multilevel modelling in attempts to differentiate these influences. But this was not the case for the studies reviewed here: a minority used multilevel models. Not using such models did not, however, prevent some authors from inferring on community-level processes, extrapolating from purely individual-level models.

In explaining the spatial patterning of disability, the distinction between spatial clustering of individual characteristics (*i.e.*, compositional effects) and contextual effects is of key importance for understanding socioenvironmental influences. The lack of distinction between multiple levels (scales) of influences can lead to inferential errors and potentially to ill-targeted policies. In one study [64], non-statistically significant estimates of local-area characteristics (derived from individual-level data) in classical OLS regression were interpreted as implying the absence of contextual effects beyond the effects of individual socioeconomic factors included in the model. Not using multilevel models was justified by a low frequency of individuals per census communities, and having controlled for inter-individual correlation due to sampling design. Not using multilevel models might be justified in the absence of group-level dependency within a data set. However, multilevel models do more than “remove” an undesirable dependency; they allow for inference on contextual influences through concurrent testing of associations of an outcome with group-level and individual-level factors. Without testing of multiple levels, one cannot simultaneously make any inference regarding individual-level and contextual influences when the latter are estimated based on aggregation of individual characteristics. Moreover, use of multilevel models expands the possibilities for investigating the person-environment interaction though cross-level effect modification testing [86]. Nonetheless, multilevel models are not a panacea: they assume within-area homogeneity and a mutual exclusivity between individual and socioenvironmental factors [83]. It is therefore essential that their use be based on conceptually founded spatial units, namely for avoiding the pitfall of over-adjustment and thereby gaining inferential validity.

## 5. Conclusions

Conceptual models of disability now generally integrate socioenvironmental influences for understanding the processes leading to disability. The papers reviewed here suggest that socioenvironmental factors are influential. A notable contribution of this review is in determining the diversity of conceptual underpinnings and operational measures of disability and socioenvironmental factors, as well as variations in a study’s methodological choices and study designs. The conjunction of these challenges precludes a straightforward synthesis in terms of effect size estimates and implications for policy making. Evidence of socioenvironmental influences on disability in the general adult population remains scarce and dispersed relative to the corpus of epidemiological literature addressing socioenvironmental influences on health outcomes.

This review has limitations. Among these is its specificity. Firstly, our search was thesaurus-dependant and hence the retrieved papers were based on the terms used for indexation. However, among the papers known to the authors to fit the inclusion criteria, only three were not retrieved by our search [87,88,89]. Supplementing our review with these papers would not have altered its overall findings. Another potential limitation is not having used synonyms for disability. We do not, however, see this as a strong threat undermining any of the interpretations or cautions expressed here given that synonyms often lead to the same thesaurus entry.

Secondly, our review did not meet all the requirements for systematic reviews. However, we believe our process led to valid results. In effect, in a similar review on neighbourhood-level influences on various health outcomes (including health-related disability) in studies targeting elderly populations, Clarke and Nieuwenhuijsen [20] obtained results similar to those from our review. Clarke and Nieuwenhuijsen observed that only a minority of studies referred explicitly to a conceptual framework and tested specific processes, that area-level SES is analysed by a large proportion of the studies and appears to act as a risk factor, that a majority of studies are cross-sectional, and that studies seldom justified the choice of geographical scale or spatial delineation of the neighbourhoods. These issues were also identified in our review.

Thirdly, our focus on epidemiological analyses may have yielded a different portrait of the literature than what one would have obtained from qualitative studies. Qualitative studies address issues not covered by the reviewed reports, such as sociospatial construction of disability [90,91], experiences of space by disabled individuals [92,93] and marginalisation caused by the restricted accessibility of the built environment [94,95]. Differences in analytic strategies between the later corpus and the epidemiological studies reviewed here preclude a detailed comparison. Nonetheless, we opine a review of qualitative analyses may have found results similar to ours. For example, Dear *et al.* [90] reported variability in the instruments used for analysing spatial variations in disability. However, some differences are also to be expected, such as the theoretical grounding [96] and the identification of specific mechanisms. For example, Butler and Bowlby [97] described the dominant medical discourse as a mediator in the construction of self-identities and Chouinard [98] demonstrates socio-spatial variations in access to legal rights. A reflection on the role of geographical scale was also offered by Kitchin and Wilton [99]. An important and fundamental difference likely to affect how socioenvironmental influences are analysed and interpreted lies in the object of study: qualitative studies tend to describe disability through individuals’ experiences of space whereas quantitative analyses focus on the statistical distributions of spatially-based risk factors within populations (*i.e.*, group of individuals). This difference corresponds to variations in how socioenvironmental factors are measured [100].

Another limitation is having included in this review only studies targeting the general population. This may have under-evaluated the total body of evidence by excluding studies of specific populations (e.g., pathology-specific groups). Also, we found no study using multi-country samples. Multi-county samples would potentially allow a greater diversity of neighbourhood-factors, which could provide different insights on the influence of such factors. We are unaware of studies which would allow to assess how multi-country samples would differ from those analysed in the studies reviewed here.

This review highlighted elements which can potentially improve research on socioenvironmental influences on the disablement process. One of these is the need for measures distinguishing between person-level abilities (context-free) and disability (context-dependent) [101]. This is a challenging task and one dependent upon conceptualisation. However, only measures making this distinction will allow isolating socioenvironmental influences.

Few of the identified studies hypothesised and tested a precise relationship or set of relationships between socioenvironmental and disability constructs presented in a disability-specific conceptual model. Even though pleas for theory-driven research were made in the health sciences [83,102,103], this result is not surprising. Studies of socioenvironmental determinants of disability are a relatively recent focus in epidemiology. Nonetheless, disability-specific models need to be tested so that knowledge can be gained on the mechanisms by which socioenvironmental factors influence the disablement process, with conceptual adjustments subsequently made as necessary. Many conceptual models consider disability as a person-environment interaction. This implies that research ought to analyse socioenvironmental factors as risk factors for individual-level capacities or as moderators or effect modifiers of (in interaction with) the effect of individual-level capacities on disability.

Contextual influences on disability are manifestations of a large set of interplaying elements (including social processes and attitudinal environments). Therefore, informing public policy requires a detailed understanding of the determinants and processes. The disablement process is made of complex interactions between many diverse elements. Policies need to be elaborated on taking into consideration of the broader social context of enablement, expanding to include domains such as social acceptance and equality, and accessibility regulations. Understanding conceptual and operational issues reviewed here can inform or assist future investigations into the role of socioenvironmental factors in the disablement process.
